# The Transcriptomes of *Xiphinema index* and *Longidorus elongatus* Suggest Independent Acquisition of Some Plant Parasitism Genes by Horizontal Gene Transfer in Early-Branching Nematodes

**DOI:** 10.3390/genes8100287

**Published:** 2017-10-23

**Authors:** Etienne G.J. Danchin, Laetitia Perfus-Barbeoch, Corinne Rancurel, Peter Thorpe, Martine Da Rocha, Simon Bajew, Roy Neilson, Elena Sokolova (Guzeeva), Corinne Da Silva, Julie Guy, Karine Labadie, Daniel Esmenjaud, Johannes Helder, John T. Jones, Sebastian Eves-van den Akker

**Affiliations:** 1 INRA, Université Côte d’Azur, CNRS, ISA, 06903, Sophia Antipolis Cedex, France; etienne.danchin@sophia.inra.fr (E.G.J.D.); laetitia.zurletto@sophia.inra.fr (L.P.-B.); Corinne.Rancurel@sophia.inra.fr (C.R.); martine.darocha@sophia.inra.fr (M.D.R.); Daniel.Esmenjaud@sophia.inra.fr (D.E.); 2 Cell and Molecular Sciences Group, Dundee Effector Consortium, James Hutton Institute, Invergowrie, Dundee DD2 5DA, UK; Peter.Thorpe@hutton.ac.uk (P.T.); simon.bajew@crg.eu (S.B.); guzeyeva@mail.ru (E.S.G.); John.Jones@hutton.ac.uk (J.T.J.); 3 Ecological Sciences Group, IPM@Hutton, James Hutton Institute, Invergowrie, Dundee DD2 5DA, UK; Roy.Neilson@hutton.ac.uk; 4 Centre of Parasitology of the A.N. Severtsov Institute of Ecology and Evolution, Russian Academy of Sciences, Leninskii Prospect 33, Moscow 119071, Russia; 5 Commissariat à l’Energie Atomique (CEA), Institut de Génomique (IG), Genoscope, 92057, Evry, France; dasilva@genoscope.cns.fr (C.D.S.); jguy@genoscope.cns.fr (J.G.); klabadie@genoscope.cns.fr (K.L.); 6 Laboratory of Nematology, Department of Plant Sciences, Wageningen University, Droevendaalsesteeg 1, 6708 PB Wageningen, The Netherlands; Hans.Helder@wur.nl; 7 School of Biology, University of St Andrews, North Haugh, St Andrews KY16 9TZ, UK; 8 Biological Chemistry, John Innes Centre, Norwich Research Park, Norwich NR4 7UH, UK; 9 School of Life Sciences, University of Dundee, Dundee, DD1 5EH, UK

**Keywords:** glycoside hydrolase, horizontal gene transfer, nematodes, plant parasitism

## Abstract

Nematodes have evolved the ability to parasitize plants on at least four independent occasions, with plant parasites present in Clades 1, 2, 10 and 12 of the phylum. In the case of Clades 10 and 12, horizontal gene transfer of plant cell wall degrading enzymes from bacteria and fungi has been implicated in the evolution of plant parasitism. We have used ribonucleic acid sequencing (RNAseq) to generate reference transcriptomes for two economically important nematode species, *Xiphinema index* and *Longidorus elongatus*, representative of two genera within the early-branching Clade 2 of the phylum Nematoda. We used a transcriptome-wide analysis to identify putative horizontal gene transfer events. This represents the first in-depth transcriptome analysis from any plant-parasitic nematode of this clade. For each species, we assembled ~30 million Illumina reads into a reference transcriptome. We identified 62 and 104 transcripts, from *X. index* and *L. elongatus*, respectively, that were putatively acquired via horizontal gene transfer. By cross-referencing horizontal gene transfer prediction with a phylum-wide analysis of Pfam domains, we identified Clade 2-specific events. Of these, a GH12 cellulase from *X. index* was analysed phylogenetically and biochemically, revealing a likely bacterial origin and canonical enzymatic function. Horizontal gene transfer was previously shown to be a phenomenon that has contributed to the evolution of plant parasitism among nematodes. Our findings underline the importance and the extensiveness of this phenomenon in the evolution of plant-parasitic life styles in this speciose and widespread animal phylum.

## 1. Introduction

Plant-parasitic nematodes (PPN) cause damage to crops across the world and are a major threat to global food security. A phylogenetic analysis of the phylum Nematoda [1,2] has shown that the ability to parasitize plants has arisen independently on at least four separate occasions within the phylum. The majority of the most economically important PPN species are located in Clade 12 (Tylenchida), and include migratory endoparasitic species as well as the biotrophic, sedentary endoparasitic root-knot and cyst nematodes. These nematodes, and the Clade 10 plant parasite *Bursaphelenchus xylophilus*, have been intensively studied and extensive genome and transcriptome resources are available for these nematodes. These resources include full genome sequences for several root-knot and cyst nematodes (e.g., [3,4,5,6]) and *B. xylophilus* [7] as well as extensive transcriptome analysis for a wide range of other species in these clades (reviewed in [8]). In contrast to endoparasitic nematodes that are restricted to Clades 12 and 10, ectoparasitic nematodes species can be found in all four plant parasite Clades [9]. However, very little genome or transcriptome information is available for the ectoparasitic nematodes in Clades 1 (e.g., Trichodoridae) and 2 (e.g., Longidoridae) other than a small-scale expressed sequence tag project for the Longidoridae *Xiphinema index* [10]. Consequently, the molecular process by which Clades 1 and 2 ectoparasitic nematodes infect plants is poorly known.

Ectoparasitic nematodes from Clades 1 and 2 cause damage to plants, either through direct feeding or by transmission of plant viruses. The economic damage caused by plant viruses explains why major vector species, including the nematodes *X. index* and *Longidorus elongatus*, are among the most studied ectoparasites. Both these nematodes belong to the family Longidoridae and are members of Clade 2 of the phylum. *Longidorus elongatus* has mainly been found in temperate regions. It feeds on a wide variety of herbaceous annual and perennial crops and weeds [11,12] and transmits two major plant viruses, Raspberry ringspot virus (RRV) and Tomato black ring virus (TBRV). *Xiphinema index* is primarily recorded on grapevine, though it can develop on other perennial crops [13]. Its native area is the Middle East, from where it has been spread with cultivated grapevine to viticulture regions globally [14]. It has a high economic impact by transmitting Grapevine fanleaf virus (GFLV), the major grapevine virus worldwide [15,16]. Both *L. elongatus* and *X. index* are diploid (with *n* = 7 and *n* = 10 chromosomes, respectively). Both reproduce by meiotic parthenogenesis [17] and rarely have males. For *X. index*, sexual reproduction has been described [18] but it is the exception, and the biotic or environmental factors that activate sexual reproduction are unknown. As for other Longidorids, multiplication of both nematodes is slow compared to Clade 12 plant parasites, and development from egg to adult may take one to several years [11,19].

All life stages of *X. index* and *L. elongatus* occur outside the host. *Xiphinema index* feeds using its long hollow odontostyle to penetrate root cells and ingest cell contents. When feeding at the root tip, large multinucleate, metabolically active cells, similar in appearance and ontogeny to the giant cells of Clade 12 root-knot nematodes, are formed [20]. At sites other than the root tip, the nematode feeds from a column of cells in the root [21,22] and can induce necrosis of damaged tissues. Responses of the host plant may also differ depending on the host status of the plant. For example, *Ficus carica* is a good host for *X. index* and modified host cells are seen frequently, whereas on a poor host (e.g., *Solanum lycopersicum*) non-modified cells are observed at root tips [20]. Feeding by *X. index* alternates phases of withdrawal and ingestion of cell contents with phases of inactivity when the nematode is thought to be injecting saliva. Feeding behaviour and gall formation by *L. elongatus* are similar, even though this nematode feeds exclusively on the root tips of its hosts and does not induce multinucleate cells because mitosis and cytokinesis occur together during hyperplasia [23]. Changes to nuclei and DNA levels within galls induced by *L. elongatus* have been recorded [24]. 

Analysis of the genomes and transcriptomes of PPN to date has shown that horizontal gene transfer (HGT) has substantially contributed to plant-parasitic nematode genomes and played a key role in the evolution of plant parasitism [8,25]. While not all genes acquired via HGT have been implicated in parasitism, and similarly not all genes implicated in parasitism are acquired via HGT, some genes acquired via HGT are indeed clearly involved in the parasitism process. For instance, a wide range of cell wall-degrading enzymes, including cellulases and pectate lyases, is present in Clade 10 and 12 PPN (reviewed in [25]). A key finding is that multiple independent HGT events have occurred and may have facilitated the evolution of plant parasitism in several different groups. This is best illustrated by the presence of different cellulases in Clade 10 and Clade 12 PPN; the Clade 12 PPN contain Glycoside Hydrolase Family 5 (GH5) cellulases that are most likely to have been acquired from bacteria [26], while the Clade 10 PPN *B. xylophilus* contains Glycoside Hydrolase Family 45 (GH45) cellulases that were probably acquired from fungi [27]. Besides degradation of the plant cell wall, some genes acquired via HGT have been shown to be involved in processing of nutrients from the plants or manipulation of plant defence system [25]. For example, root-knot and cyst nematodes have acquired invertases from bacteria that convert sucrose from the plant into glucose and fructose, readily processed by animals [28].

The absence of genome and transcriptome information for early-branching ectoparasitic nematodes means that it is not known whether HGT has also been important in the evolution of plant parasitism in these groups. In order to better understand the mechanisms underpinning parasitism by these ectoparasitic nematodes, and to determine the extent of HGT in these independently evolved PPN, we report the deep sequencing of transcriptomes of *X. index* and *L. elongatus* and an analysis of genes potentially acquired via HGT in these species. We demonstrate the presence of a biochemically active Glycoside Hydrolase Family 12 (GH12) cellulase of likely bacterial origin in *X. index*, confirming that independent HGT has occurred in this group and may have played a role in the evolution of plant parasitism.

## 2. Materials and Methods

### 2.1. Sample Preparation and Sequencing

Mixed developmental stages of *X. index*, reared in a greenhouse on fig plants, were collected and pooled together for RNA extraction and complementary DNA (cDNA) library preparation. To maximize the number of expressed genes, we produced *X. index* specimens both in standard and stress conditions. Stressed *X. index* were obtained by soaking for 1 h in 0.2 M NaCl, for 30 min in 1% EtOH, and for 1 h at 40 °C or for 1 h in 0.1% acetic acid. Nematodes from stress treatments were pooled before RNA extraction. Total RNA was extracted using TRIzol reagent (Invitrogen, Carlsbad, CA, USA) and reverse transcription was carried out using the Ovation pico WTA System (NuGEN Technologies, Inc., San Carlos, CA, USA). The two libraries, non-stressed and stressed, were sequenced on an Illumina GAIIx sequencer (Illumina, San Diego, CA, USA) to produce 76 nucleotide (nt) single end reads.

Mixed stages of *L. elongatus* were collected from soil samples from various farms in Scotland and identified visually under a binocular microscope before being frozen in liquid nitrogen. *Longidorus elongatus* were hand-picked from samples that were previously identified under a microscope as *L. elongatus*. No other *Longidorus* species were present from the site sampled. RNA was extracted using an RNEasy Plant Mini kit (Qiagen, Crawley, UK) following the manufacturer’s instructions with the on-column DNAse digestion. Sequence was generated on an Illumina MiSeq (Illumina, San Diego, CA, USA) to produce 150 basepair (bp) paired-end reads using standard protocols.

All raw RNA sequencing (RNAseq) data produced for the research described in the manuscript is available under the Sequence Read Archive (SRA) accession numbers PRJEB8328 and PRJEB22758 for *L. elongatus* and *X. index*, respectively.

### 2.2. Reads Quality Control and Transcriptome Assembly

Read quality, length and composition were checked using FASTQC (http://www.bioinformatics.babraham.ac.uk/projects/fastqc/). For *X. index*, ribosomal RNAs were detected and removed using SortMeRNA [29]. PRINSEQ [30] was used to remove sequences with a minimum quality score of 30 and less than 50 nucleotides long. Cleaned reads were assembled de novo using Trinity v2.2.0 [31] with 4 different *k*-mer sizes (22, 24, 25 and 26). We concatenated the 4 assemblies and eliminated redundancy using MegaBLAST [32] and CAP3 [33]. Reads were mapped back to the *X. index* assembly and normalized expression for each transcript in each library was calculated using the Trinity wrapper scripts for edgeR [34] and RSEM (https://deweylab.github.io/RSEM/) (Supplementary Table S1). For *L. elongatus*, 150 bp paired-end reads were trimmed of bases with low support (Phred score < 30), and cropped to a minimum length of 50 nucleotides and a maximum length of 100 nucleotides to remove non-random base distributions. Paired and unpaired reads which passed the quality criteria were concatenated and used for de novo assembly using Trinity v2.2.0 using a *k*-mer size of 25 and a *k*-mer coverage of 3. Transcripts in the resulting assembly were scored based on the left and right read pair mapping characteristics using Transrate [35], and low supported transcripts were removed in two iterations. We calculated N50 to measure contiguity and used BUSCOv2 [36] to evaluate completeness based on the dataset of 978 groups of proteins highly conserved in Metazoa.

### 2.3. Identification of *L. elongatus* GH12

To identify the *L. elongatus* GH12 sequence, a draft low coverage assembly was generated using Trinity with a minimum *k-*mer coverage of 1. The *X. index* GH12 was used as query in BLAST to identify a GH12 fragment. The 5′ and 3′ ends of this transcript fragment were computationally extended using an iterative approach: raw reads overlapping with a minimum *k-*mer of 26 nucleotides were retrieved and assembled to extend the fragment using MITObim_1.6 (https://github.com/chrishah/MITObim). Four rounds of this extension produced a complete GH12 transcript.

### 2.4. Open Reading Frame Prediction

Open Reading Frames (ORFs) were predicted in both transcriptomes using a custom pipeline developed to more faithfully predict 5′ start codons. Initially, TransDecoder 2.0.1 [31] was used to predict ORFs encoded by the assembled contigs, with the modification that the hard-coded value within the TransDecoder script for redundancy removal of 80% was changed to 99%. BLASTp results are used by TransDecoder when determining the coding sequence: the SwissProt database (September 2015) including the gene models for *Globodera pallida* [5], *G. rostochiensis* [6] and *B. xylophilus* [7] were used as reference. Pfam A and B domain definitions (2014) were also used to guide TransDecoder coding sequence (CDS) prediction. The final TransDecoder predicted coding sequences were subjected to a 5′ CDS refinement tool, available at: https://github.com/peterthorpe5/public_scripts/tree/master/Fix_five_prime. This tool compares the average coverage per nucleotide of the starting codon to the average coverage of the middle 50% of the coding sequence. If the starting position is less than five standard deviations from the mean coverage (a user-defined threshold) then this is classed as an untranslated (UTR) region. The tool then finds the next ATG start codon and re-tests the coverage based on the same logic process until a suitable start is found.

### 2.5. Prediction of Groups of Orthologs

We used OrthoFinder v1.1.4 [37] to identify groups of orthologs and in-paralogs (orthogroups) between *L. elongatus*, *X. index* and other nematodes. We compared the predicted proteomes of *L. elongatus* and *X. index* produced in this study to those of 10 other nematodes: the Clade 1 animal parasite *Trichinella spiralis* [38]; three Clade 12 apomictic plant-parasitic root-knot nematodes *Meloidogyne incognita*, *M. javanica* and *M. arenaria* [39] as well as the meiotic facultative parthenogens *M. hapla* [4] and *M. floridensis* [40]; the two Clade 12 plant-parasitic cyst nematodes *Globodera pallida* [5] and *G. rostochiensis* [6]; the Clade 10 plant-pathogenic nematode *B. xylophilus* [7]; and the Clade 9 model free-living bacterivorous nematode *C. elegans* [41]. All the proteomes were compared against each other and themselves and orthogroups were inferred based on reciprocal best and better hits and Markov Cluster Algorithm (MCL) clustering.

### 2.6. Pfam and Gene Ontology Annotation

We used PfamScan (available at ftp://ftp.ebi.ac.uk/pub/databases/Pfam/Tools/) to predict conserved protein domains and repeats in the *X. index* and *L. elongatus* predicted proteins. Each protein was scanned against the Pfamv30 collection of Hidden Markov Models (HMMs) [42] using PfamScan default parameters and with a prediction of active sites. We used the pfam2GO association file maintained at http://geneontology.org/external2go/pfam2go to assign gene ontology terms based on the presence of predicted Pfam domains in proteins. We used the hypergeometric test implemented in FUNC [43] with a False Discovery Rate (FDR) threshold of 0.05 to detect gene ontology terms significantly enriched in genes putatively acquired via HGT.

### 2.7. Prediction of Candidate Horizontal Gene Transfer

Putative HGT events in *X. index* and *L. elongatus* were detected by the Alienness software [44,45] which calculates an Alien Index (AI) as initially described in [46]. Briefly, all predicted proteins were compared against the National Center for Biotechnology Information (NCBI) non-redundant library (nr) using BLASTp [32] with an E-value threshold of 1E^−3^ and no low-complexity filtering. BLAST results were parsed to retrieve associated taxonomic information, using the NCBI’s taxonomy as a reference. For every protein returning at least one hit in either a metazoan or non-metazoan species, an AI was calculated according to the following formula:

$$AI=\;\ln\left(best\;metazoan\;E-value+\;1E^{-200}\right)-\ln\left(best\;non\_metazoan\;E-value+\;1E^{-200}\right).$$

When neither a metazoan nor non-metazoan BLAST result was found, a penalty E-value of 1 was automatically assigned as the best metazoan or non-metazoan E-value, respectively. To allow detection of HGT events that took place in an ancestor of *X. index* and *L. elongatus*, (self) BLAST results to Longidoridae (TaxID: 46001) were skipped for the calculation of AI. No AI value could be calculated for proteins returning no significant hit at all in nr. An AI > 0 indicates a better hit to a non-metazoan species than to a metazoan species and possible acquisition via HGT of non-metazoan origin. We recently showed that with an AI > 9, all known HGT cases in PPN supported by phylogenetic analysis are retrieved [45] but there is still some risk for possible false positives at this threshold. To focus on high-confidence candidates and minimize the risk of false positives, we selected as threshold an AI > 30, which corresponds to a difference of magnitude 1E^14^ between the best non-metazoan and best metazoan E-values. An AI > 30 was also shown as a good balance between sensitivity and specificity for Alien Index scores on various nematodes [6,45,47] and other organisms [48]. All proteins that returned an AI > 0 and that aligned with ≥70% identity to a non-metazoan protein were considered as putative contaminants and were discarded from the analysis.

### 2.8. GH12 Cellulase Cloning and Functional Characterization

Part of a GH12 cellulase sequence was originally identified in two expressed sequence tags (ESTs) from a cDNA library made from mixed stages of *X. index* [10]. Analysis of these sequences (accession numbers CV508600.1 and CV127906) suggested that the sequenced part of the gene was incomplete at the 5′ end. Rapid amplification of cDNA ends (RACE) was therefore used to clone the remaining portion of this gene using standard protocols. For biochemical testing, the full-length sequence, with the signal peptide removed, was cloned into a bacterial plasmid expression vector and cellulase activity of the proteins produced was tested using carboxymethylcellulose as a substrate as previously described [49].

### 2.9. Phylogenetic Analyses

We used the *X. index* GH12 predicted protein as a BLASTp query against the NCBI nr library with an E-value threshold of 0.01 and a maximum number of target sequences of 250. We retrieved the 250 best BLAST results and added two *X. americanum* sequences that we identified in a genome skimming analysis [50] as well as the one from *L. elongatus*. We removed redundancy at 90% identity using CD-HIT [51] and aligned the remaining 128 non-redundant sequences using MAFFT [52] with automatic selection of the most appropriate alignment strategy. We eliminated alignment columns that contained more than 50% of gaps, using TrimAl [53]. We performed a maximum likelihood (ML) phylogeny with RaXML v8.2.9 [54], using an automatic selection of the fittest evolutionary model and a gamma distribution of the rates of evolution. We used the autoMRE option to automatically stop bootstrap replicates upon convergence. The Maximum Likelihood (ML) phylogenetic analysis finished after 550 bootstrap replicates, and the fittest evolutionary model was the Le and Gascuel (LG) model [55]. We performed a Bayesian phylogeny on the same multiple alignment with MrBayes 3.2.6 [56], using an estimated gamma distribution of rates of evolution as well as the proportion of invariable sites and an automatic selection of the most probable evolutionary model. We ran 3,850,000 Markov Chain Monte Carlo (MCMC) generations on eight chains and the simulations converged to an average deviation of split frequencies < 0.048. The most probable evolutionary model was the Whelan and Goldman (WAG) model [57]. Because this first phylogenetic analysis did not allow discovery of which bacterial proteins were the most closely related to GH12 sequences of the Clade 2 plant-parasitic nematodes, we performed a second analysis focused only on the 100 best BLAST results. We used the same strategy for the elimination of redundancy, cleaning of the alignment and ML and Bayesian phylogenies. The ML phylogeny converged after 850 bootstrap replicates and the fittest evolutionary model was also LG. The Bayesian phylogeny was run using 1,000,000 MCMC generations. The average deviation of frequency was < 0.02 and we discarded the initial 25% of trees to calculate probabilities and establish the final tree. The WAG model was found to be the most probable evolutionary model. We midpoint-rooted, visualized and coloured all the trees with FigTree v1.4.3 (http://tree.bio.ed.ac.uk/software/figtree/).

## 3. Results

We sequenced, assembled and analysed the mixed-stage transcriptomes of two migratory ectoparasites from Clade 2: *X. index* and *L. elongatus*. Plant parasites in this clade are relatively underexplored, and thus these data afford an opportunity to explore their unique biology.

### 3.1. Sequencing and Transcriptome Assembly

For *X. index*, approximately 29 million 76 bp single reads were produced for each of two libraries (standard conditions and stressed conditions, Table 1). Both libraries were pooled and after quality control a total of ~36 million reads were used for de novo assembly and subsequent analyses. The assembly yielded 48,920 contigs and returned N50 value of 1012 nucleotides with shortest and longest sequences lengths of 201 and 19,593 nucleotides, respectively. Full length transcripts of 73.8% of the 978 evolutionarily conserved BUSCO Metazoan genes were identified, with a further 17% present as partial transcripts (only 9.2% of BUSCO genes were not found in the assembly).

For *L. elongatus*, approximately 39 million 150 bp paired end reads were produced from mixed stage nematodes (Table 1). Following quality control, surviving paired and unpaired reads were concatenated, producing a total of approximately 30.5 million reads (~27.5 million pairs) used for de novo assembly and further analysis. The final assembly of 57,954 contigs had N50 of 1182 and a shortest and longest contig length of 201 and 13,675 nucleotides, respectively. Full-length transcripts of 77.7% of the 978 evolutionarily conserved BUSCO Metazoan genes were identified, with a further 10.9% present as partial transcripts (only 11.4% of BUSCO genes were not found in the assembly). The assembled transcriptomes of *L. elongatus* and *X. index* are thus largely comparable in overall size and completeness.

### 3.2. Protein Prediction, Annotation and Contamination Removal

ORFs were predicted in both transcriptomes using a custom pipeline (see methods for details). An initial set of 25,795 and 14,759 ORFs were predicted for *L. elongatus* and *X. index*, respectively, using TransDecoder. We used an in-house read-coverage based correction method to redefine start codons for 1555 ORFs for *L. elongatus* (6%) and 42 ORFs for *X. index* (0.3%).

ORFs with an AI >0 (i.e., more resembling a non-metazoan protein) and displaying at least 70% identity to the best non-metazoan hit, were considered putative contaminants, and removed from further analyses. We found 76 and 57 such ORFs in the *L. elongatus* and *X. index* transcriptomes, respectively (Supplementary Table S2). The majority of putative contaminants in the *L. elongatus* transcriptome (40/76) were of viral origin with 29 sequences from the Beet ringspot virus, six from the Tomato black ring virus (STRAIN L), two from Tomato black ring virus (no strain indicated), two from Turnip yellow virus and one from white clover cryptic virus. Of these 40 sequences, the first 37 listed are derived from Nepoviruses that are known to be vectored by *Longidorus* species. In contrast, only one sequence out of 57 *X. index* putative contaminants was of viral origin (Tobacco mosaic virus strain tomato/L). Given that *X. index* specimens were reared on fig trees before transcriptome sequencing, where they lose their viral load [58], whereas *L. elongatus* were extracted from agricultural field soil, the presence of viral sequences in the *L. elongatus* transcriptome may have biological relevance.

Read coverage-corrected ORFs were annotated using Pfam v30 [42]. A total of 3415 and 3157 different Pfam domains were identified in the predicted proteins of *L. elongatus* and *X. index*, respectively. More than 80% of these domains (2774) were conserved in both species, indicating that the predicted protein sets of these two species encode a similar variety of domains. Based on the presence of Pfam domains, a total of 1059 and 1001 different Gene Ontology (GO) terms were assigned to predicted proteins in *L. elongatus* and *X. index*, respectively. Similar to Pfam domains, more than 88% of the GO terms were common to both species.

### 3.3. Comparisons with Other Nematodes

We clustered the predicted proteins of *L. elongatus* and *X. index* together with those from 10 other nematodes in groups of orthologs and in-paralogs (orthogroups, cf. methods). For inclusion in this comparative analysis, we selected 7 plant-parasitic nematodes from Clade 12, one from Clade 10, an animal parasite from Clade 2, and the free-living model *C. elegans* from Clade 9. Overall, the proteins of the 12 nematodes were clustered in 31,834 orthogroups. We found that 21,468 (83.3%) and 11,561 (78.3%) *L. elongatus* and *X. index* proteins, respectively, were assigned to orthogroups containing one or more other nematode species. Conversely, 4298 *L. elongatus* (16.7%) and 3198 *X. index* (21.7%) proteins were not assigned to any orthogroup and represented species-specific singleton proteins, while a further 111 *L. elongatus* (440 proteins) and 66 *X. index* (171 proteins) orthogroups were species-specific multicopy proteins. Species-specific multicopy proteins are those represented by more than one sequence in the transcriptome and does not distinguish between isoforms and gene families. We identified 1660 orthogroups that were unique to the Clade 2 nematodes (*T. spiralis*, *L. elongatus* and *X. index*), of which 187 orthogroups were conserved in the three species and could represent core Clade 2-specific proteins. We also identified 1108 orthogroups that were unique to *L. elongatus* and *X. index* and conserved in these two PPN. These orthogroups covered 2664 and 1549 proteins from *L. elongatus* and *X. index*, respectively. This dataset represents an interesting group of proteins that may contain genes specifically associated with the evolution of plant parasitism in Clade 2 nematodes.

We performed a similar comparative analysis at the Pfam domain level with the same 12 nematode predicted proteomes. We identified 437 Pfam domains that were uniquely found in at least one Clade 2 nematode but absent from the other nematodes investigated (Supplementary Table S3). Among these, 45 Pfam domains were conserved in the three studied Clade 2 nematodes (Supplementary Table S4). These Pfam domains might correspond to core functions specific to Clade 2 nematodes or to domains that have been secondarily lost in the other nematodes. To identify Clade 2 proteins that are putatively involved in plant parasitism, we pinpointed 91 Pfam domains that were uniquely present in both *X. index* and *L. elongatus* (Supplementary Table S5).

### 3.4. Detection of Candidate Horizontal Gene Transfer Events 

ORFs with an AI > 30 (i.e., much more similar to a non-metazoan protein than a metazoan protein) and displaying less than 70% identity to their most similar non-metazoan sequence, were considered putatively acquired by HGT. We found 104 and 62 predicted proteins in *L. elongatus* and *X. index* that respectively fulfil these criteria (Supplementary Table S6). Over half of these, 64/104 and 35/62 were clustered in 22 and 25 orthogroups, respectively. All but two of these orthogroups contained more than one nematode species, suggesting that the transfer events were either ancestral to the separation of the different nematodes or that they were acquired multiple times independently.

We identified 13 orthogroups containing HGT candidates that were common to *L. elongatus* and *X. index*, but not the Clade 2 animal parasite *T. spiralis*. Nine of these were restricted to the available *X. index* and *L. elongatus* transcriptome data, and may indicate either transfer events ancestral to the separation of *L. elongatus* and *X. index* but after their divergence from *T. spiralis*, or early acquisition in Clade 2 and secondary loss in *T. spiralis*. The remaining four orthogroups correspond to more complex possible acquisitions. One case encompassed one *X. index* and two *L. elongatus* proteins bearing a GH32 ‘Glyco_hydro_32N’ domain (PF00251.18). These Clade 2 GH32 proteins were clustered together with GH32 of Clade 12 plant-parasitic nematodes, root-knot and cyst nematodes, for which an invertase activity has been biochemically characterised and an acquisition via HGT supported by a phylogenetic analysis [28]. Invertases catalyse the conversion of sucrose to glucose and fructose, and could thus be involved in assimilation of plant sugar by the nematodes. Interestingly, the GH32 proteins of the Clade 2 plant-parasitic nematodes had most similar BLAST results to those of Bacteroidetes (avg. 45% identity). This is in contrast with the GH32 proteins of Clade 12 nematodes that are more closely related to Rhizobial bacteria [28]. These findings suggest two independent acquisitions in Clade 2 and Clade 12 plant-parasitic nematodes rather than a single common acquisition in an ancestor of all these nematodes.

Interestingly, some Pfam domains were specific to proteins putatively acquired via HGT. We identified 11 *L. elongatus* and 11 *X. index* Pfam domains that were only present in predicted proteins with an AI >30; strongly indicative of possible acquisition via HGT (Table 2). Four of these Pfam domains were common to both *L. elongatus* and *X. index*. This included the Glyco_hydro_32N (candidate invertases) discussed above as well as DUF2238, DOPA_dioxygen and a Lactanase domain. 

Based on the Pfam annotation, we assigned GO terms to the predicted proteins of both nematodes and we searched for significant GO enrichment in the proteins having an AI > 30 (HGT candidates). In the ‘Molecular Function’ ontology, seven and 23 GO terms were enriched in candidate HGT of *X. index* and *L. elongatus*, respectively (Supplementary Table S7). One GO term (ammonium transmembrane transporter activity) was common to both species. In the ‘Biological Process’ ontology, 12 and 46 GO terms were enriched in candidate HGT of *X. index* and *L. elongatus*, respectively. Two terms were common to both species (ammonium transport; organonitrogen compound metabolic process). Finally, in the cellular component category, only one term was significantly enriched in HGT candidates and it was the same in both species (DNA-directed RNA polymerase II, core complex).

Among the HGT-specific Pfam domains that we found in only one of the two Clade 2 PPNs, we identified two of particular interest regarding plant parasitism. One, in the *L. elongatus* transcriptome is pantoate ligase (PF02569.13), a domain usually found in bacterial enzymes involved in biosynthesis and salvage pathway of vitamin B5. This vitamin is essential to all life and most animals have lost the capacity to synthesise this and other essential vitamins and so must take them up from their diet. Interestingly, cyst nematodes (Clade 12) have acquired enzymes from bacteria for the salvage and biosynthesis of essential vitamins, including an enzyme bearing a pantoate_ligase domain for vitamin B5 [59,60]. Pantoate ligase proteins in *L. elongatus* resemble those found in the betaproteobacteria genus *Burkholderia* (the 100 best BLAST results are all sequences of *Burkholderia* species) while those from Clade 12 nematodes resemble enzymes from Actinobacteria [59]. This suggests that two independent acquisitions of these enzymes putatively involved in biosynthesis of vitamin B5 occurred in Clade 2 and Clade 12 nematodes (similar to the GH32).

One *X. index* predicted protein contains a Glyco_hydro_12 (PF01670) domain. This domain is present in Archaea, Bacteria and Fungi but not in Metazoa (including nematodes). Enzymes bearing a Glyco_hydro_12 (or GH12) domain can have cellulase activity. Cellulases acquired via horizontal gene transfers in Clade 12 nematodes are thought to be involved in the degradation of plant cell walls and thus are directly linked to their plant-parasitic lifestyle [25,26,61]. However, cellulases of Clade 12 PPN belong to the totally unrelated GH5 family. No GH12 cellulase was found in the initial assembly of the *L. elongatus* transcriptome. To address this apparent absence, we generated an additional draft assembly for *L. elongatus* using lower minimum *k*-mer coverage. A GH12-like fragment was identified using the *X. index* GH12 as BLAST query and computationally extended to reconstitute a full length GH12 from the *L. elongatus* raw reads (methods). The corresponding *L. elongatus* GH12 protein has an AI of 114.03, and was retrospectively added to Table 2.

### 3.5. Characterisation of a GH12 Cellulase from *X. index*

**Figure 1:**
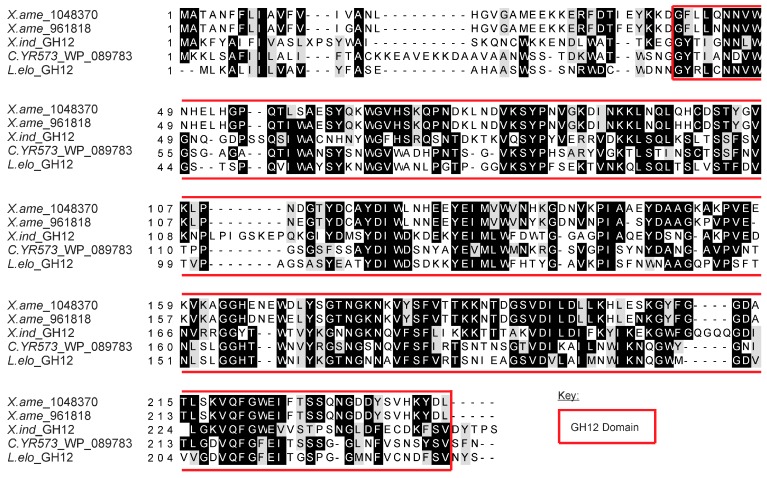
Alignment of amino acid sequences of GH12s identified from *X. index* and *L. elongatus* RNA sequencing (RNAseq), the two GH12 sequences identified from the *X. americanum* genome, and the most closely related sequence in non-redundant (nr). The GH12 domain is highlighted by a red box, and covers the majority of the protein sequence.

The *X. index* and *L. elongatus* GH12 proteins have AIs of 38.05 and 114.03, respectively, confirming their similarity to non–metazoan cellulases and suggesting acquisition via HGT. Part of a GH12 cellulase sequence was previously identified in two ESTs from a cDNA library made from mixed stages of *X. index* [10]. Analysis of these sequences (accession numbers CV508600.1 and CV127906) suggested that they are incomplete at the 5′ end, and have a poly A tail at the 3′ end. The corresponding full-length nucleotide sequence was cloned by 5′ RACE and encoded a 256 amino acid protein with a 19 amino acid eukaryotic signal peptide at its N-terminus [62]. Taken together, these attributes suggest that the GH12 identified is a nematode gene, not a contaminant transcript. In further support, a genome skim of the related plant-parasite *X. americanum* prepared independently [50] contains two similar GH12 sequences that are each encoded by a single open reading frame (Figure 1). The predicted amino acid sequence of *X. index* and *X. americanum* genes includes a complete glycosyl hydrolase family 12 domain (Figure 1).

**Figure 2:**
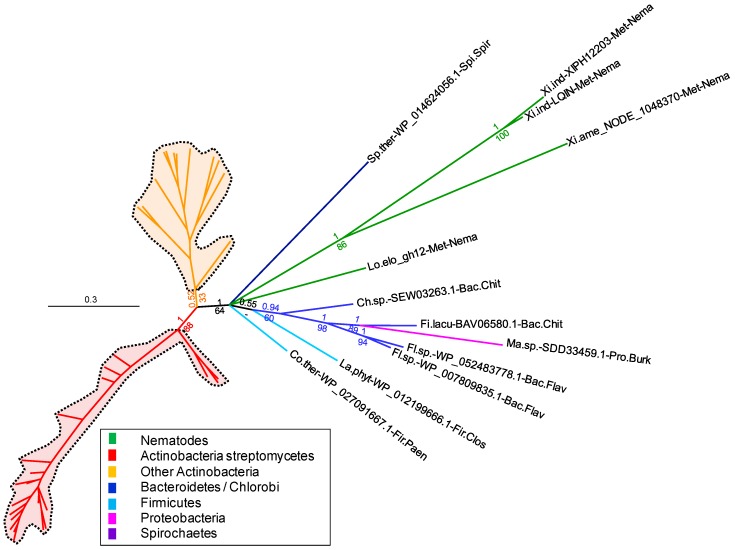
Phylogenetic tree of *X. index*, *X. americanum*, and *L. elongatus* GH12 protein and homologs. The Bayesian topology has been chosen as a reference and posterior probability support values are indicated at corresponding branches. The corresponding bootstrap support values from the Maximum Likelihood analysis are indicated below probability values. Abbreviated species names (prefix), accession number and taxonomic classification (suffix) are indicated in leaves. Full names are listed in Supplementary Table S8. The corresponding expanded phylogenetic tree is available as Supplementary Figure S2.

Using the *X. index* full-length GH12 sequence as a query confirms that the top 250 most similar proteins in the NCBI’s nr library are from bacteria, with the best BLAST hit being from *Chitinophaga* sp. *YR573* at 43% identity and an e-value of 2E^-49^ (Supplementary Table S8). An initial phylogeny performed with the top 250 BLAST results (Figure S1) shows that the *X. index* and *X. americanum* GH12 sequences form a highly supported monophyletic group in the Bayesian analysis (posterior probability, PP = 0.95). This monophyly of *Xiphinema* sequences is confirmed in the ML phylogeny too, albeit with a lower support (bootstrap = 63). Curiously, the *L. elongatus* GH12 sequence does not form a monophyletic group with the *Xiphinema* ones (neither in the Bayesian nor in the ML topologies). In both the Bayesian and ML phylogenetic analyses, a branch groups the *Xiphinema* and *L. elongatus* sequences together with GH12s of various different Bacteroidetes, Firmicutes, Proteobacteria and Spirochaetes and excludes the GH12s of Actinobacteria. This branch is supported by a moderately high posterior probability of 0.77 and a low bootstrap of 15. Because of this moderate to low support, it was not possible to confidently ascertain which group of bacteria is more closely related to the *Xihinema* and *L. elongatus* sequences under consideration. We thus performed a second phylogeny with only the 100 best BLAST results. This phylogenetic analysis globally confirms the topology of the one performed with the 250 best BLAST hits although with much higher branch support values (Figure 2 and Figure S2). The branch grouping the *Xiphinema* and *L. elongatus* GH12s with those of Bacteroidetes, Firmicutes, Proteobacteria and Spirochaetes is now supported by a posterior probability of 1 and a bootstrap of 64. This confirms with much higher support that the nematode GH12 sequences are more closely related to those of these bacteria and distinct from those of Actinobacteria. However, there is no further highly supported branch within the clade grouping the nematode GH12s to determine which group of bacteria appears to be the most closely related. Analysis of the BLAST results shows that the *X. index*, *X. americanum* and *L. elongatus* GH12s have between 38% and 42% identity to bacterial GH12s, with a query coverage ranging between 87% and 98% of the length. Overall, the phylogenetic analyses confirm a likely acquisition via HGT of bacterial origin, although identification of the precise bacterial group of the putative donor is not currently possible.

**Figure 3:**
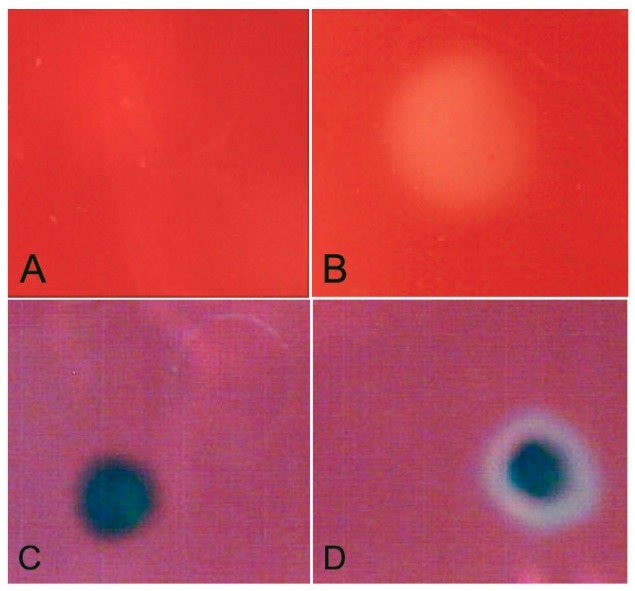
Cellulase activity of *X. index* homogenate (**B**) and the recombinant *X. index* GH12 (**D**). Negative controls are shown in panels (**A**) (buffer alone) and **C** (bacteria lacking cloned GH12 sequence).

We next analysed the capacity of *X. index*, and the identified GH12 protein, to metabolise cellulose. Homogenate of *X. index* has cellulase activity, as determined using an assay on carboxymethyl cellulose (Figure 3A,B). To test the ability of the GH12 identified from *X. index* to contribute to this activity, we cloned the predicted open reading frame (lacking the signal peptide) into a vector for heterologous protein expression in bacteria. Lysates of *E. coli* expressing the *X. index* GH12 showed biochemical activity in a cup plate assay using carboxymethylcellulose as a substrate (Figure 3C,D). Control lysates did not show this activity. Taken together this suggests that the GH12 identified is a functional cellulase, and that it may contribute to the cellulase activity of the nematode.

## 4. Discussion

Nematodes have evolved the ability to parasitize plants on at least four independent occasions: these include plant-parasites present in Clade 1 (Trichodoridae), Clade 2 (Longidoridae including *X. index* and *L. elongatus*), Clade 10 (Parasitaphelenchidae—*Bursaphelenchus* spp.) and Clade 12 (many economically important species including root-knot and cyst-forming nematodes) [1,2]. Clade 1 nematodes have not yet been examined at a molecular level due to the difficulties of obtaining biological material in the required quantities; most species in this group are microscopically small and extremely difficult to culture. However, with the addition of this study, PPN in Clades 2, 10 and 12 have now been analysed at a large-scale sequence level. In each case, the plant-parasitic nematodes have been shown to contain genes encoding cell wall degrading enzymes, underlining the importance of this process to the ability to feed on plants [25]. In all three clades, these genes have been acquired via independent HGT from a different source. For instance, GH5 cellulases are present in Clade 12 nematodes that were most likely acquired from bacteria; *Bursaphelenchus* spp. and *Aphelenchoides besseyi* (both Clade 10) contain GH45 cellulases that were most likely acquired from a fungal species associated with pine trees [63,64]. The data presented here suggest that *X. index*, *X. americanum* and *L. elongatus* contain GH12 cellulases that are likely to have been acquired from bacteria. It was not possible to precisely define which group of bacteria was a probable donor for nematode GH12s because of the absence of a highly supported branch grouping a single group of bacteria to those of these Clade 2 nematodes. This is probably because nothing resembling the donor bacteria is present in the NCBI’s nr protein library. This is not surprising, considering that bacteria from soil samples possibly inhabited by these nematodes are still underrepresented in protein libraries. However, the data we present here further emphasise the importance of the role of HGT in the evolution of plant parasitism by nematodes. The fact that an *L. elongatus* GH12 sequence was only identified in a low coverage draft assembly may be because expression of the gene is induced by exposure to a potential food source and, since the *L. elongatus* was collected from fields post-harvest and prior to planting of a crop, these nematodes were unlikely to have been actively feeding. Nevertheless, the presence of the *L. elongatus* GH12, coupled with the phylogenetic analyses, suggest a bacterial origin both in *X. index* and *L. elongatus*. 

Although *X. index* is a migratory ectoparasite, its development is associated with the formation of multinucleate, metabolically active cells adjacent to the cells located on its stylet route. This feature is reminiscent of those induced by root-knot nematodes, sedentary endoparasites from Clade 12. These modified cells are induced by the injection of saliva during feeding [21]. This particular biology suggests that, like root-knot nematode, *X. index* probably feeds indirectly from these cells. Cellulases as well as other cell-wall-degrading enzymes might be necessary to achieve this feeding style. The nematode would not only feed off the content of successive cells at increasing depths [22] but also and mainly from the nutrients transferred from the metabolically active neighbouring cells up to its stylet aperture. In fact, in ryegrass galls induced upon *L. elongatus* parasitism, ultrastructural studies have shown the presence of many empty cells with perforated cell walls [23]. It was suggested that these perforations result from the action of the nematode saliva and that their formation eases removal of the content of cells distant from the stylet tip.

Besides degradation of the plant cell wall by cellulases and other enzymes, genes acquired via HGT in plant-parasitic nematodes have been suspected to be involved in other important parasitic functions. For instance, horizontal acquisition of enzymes involved in processing of nutrients from the plant, detoxification of plant toxic compounds, and manipulation of plant defence have been reported [25]. Interestingly, in addition to the GH12 cellulase described above, both *X. index* and *L. elongatus* contain a candidate GH32 invertase. Similar genes have been identified in other PPN and their presence here may reflect a requirement for sucrose metabolism, the major translocation carbohydrate in plants. In addition, a candidate pantoate ligase, previously described in Clade 12 cyst nematodes, was identified in *L. elongatus*. Both of these enzyme classes are typically absent from metazoans and yet are present in PPN in Clades 2 and 12 of the phylum. It is interesting to note that, in all cases, while different enzymes are probably involved in the same processes (cell wall degradation and nutrient processing from the plant), in both Clades 2 and 12 plant-parasitic nematodes they probably do not originate from a common ancestral origin in nematodes. Indeed, in the case of the cellulase, the ones identified from *X. index*, *X. americanum* and *L. elongatus* belong to the family GH12, which is evolutionarily unrelated to the cellulases of family GH5 identified in Clade 12 plant-parasitic nematodes. In addition, for both the GH32 and candidate pantoate ligase, the likely donors to Clade 2 and Clade 12 nematodes are from totally different and evolutionary distant bacterial groups. This suggests several independent acquisitions of these enzymes from different sources and convergent recruitment for the same processes.

These observations reinforce the idea that, in addition to convergent morphological adaptations to plant parasitism, the acquisition of genes by HGT might be a universal adaptive feature in plant-parasitic nematodes [65]. Further exploration of nematode genomes and transcriptomes will allow for an assessment of how universal and essential this evolutionary phenomenon is for the emergence of a plant-parasitic lifestyle.

## Figures and Tables

**Table 1:** Read statistics of *Xiphinema index* and *Longidorus elongatus* transcriptome sequencing.

Library	Reads/Contigs
*X. index* standard	29,371,297
*X. index* stress	29,253,145
*X. index* Concatenation	58,624,442
*X. index* rRNA cleaning ^1^	37,280,434
*X. index* Quality filtering	21,344,008
*X. index* assembled contigs	48,920
*L. elongatus*	39,285,886
*L. elongatus* Quality filtering- paired only	27,448,536
*L. elongatus* Quality filtering- unpaired only	3,069,036
*L. elongatus* Concatenation	30,517,572
*L. elongatus* assembled contigs	57,954

^1^ *X. index* RNA was not poly-A selected and residual ribosomal RNA (rRNA) had to be removed.

**Table 2:** Pfam domains only present in Horizontal Gene Transfer (HGT) candidates of *X. index* and *L. elongatus* (numbers refer to occurrences of domains, not transcripts).

Pfam	Description	*X. index*	*L. elongatus*
PF00251.18	Glyco_hydro_32N	1	2
PF08883.9	DOPA_dioxygen	1	4
PF09997.7	DUF2238	1	6
PF10282.7	Lactonase ^1^	1	9
PF01670.14	Glyco_hydro_12	1	1 ^2^
PF00910.20	RNA_helicase	1	0
PF02569.13	Pantoate_ligase	0	3
PF03155.13	Alg6_Alg8	1	0
PF03385.15	DUF288	0	1
PF03561.13	Allantoicase	0	8
PF04982.11	HPP	0	1
PF05001.11	RNA_pol_Rpb1_R	69 ^3^	0
PF05162.11	Ribosomal_L41	1	0
PF08031.10	BBE	1	0
PF08244.10	Glyco_hydro_32C	0	2
PF13537.4	GATase_7	1	0
PF13604.4	AAA_30	0	2
PF13673.5	Acetyltransf_10	0	1

^1^ a 10th *L. elongatus* predicted protein contains a Lactonase domain with an Alien Index (AI) < 30 (AI = 21.4); ^2^ while not present in the transcriptome assembly, an *L. elongatus* GH12 cellulase transcript was reconstituted from the raw reads using MITOBIM AI > 30 (AI = 114.03); ^3^ these 69 domains are encoded by two transcripts (34 and 35 iterations of 14 amino acid domain each).

## Supplementary Materials

The following are available online at www.mdpi.com/2073-4425/8/10/287/s1. Figure S1: GH12-250-expanded-Phylogeny. Expanded phylogenetic trees of the *X. index*, *X. americanum* and *L. elongatus* GH12 cellulase sequences and their corresponding 250 best blast hits. Page 1 (A): Bayesian topology obtained with MrBayes; Page 2 (B): Maximum Likelihood topology obtained with RAxML, Figure S2: GH12-100-expanded-Phylogeny. Expanded phylogenetic trees of the *X. index*, *X. americanum* and *L. elongatus* GH12 cellulase sequences and their corresponding 100 best blast hits. Page 1 (A): Bayesian topology obtained with MrBayes; Page 2 (B): Maximum Likelihood topology obtained with RAxML, Table S1: Normalized expression of *X. index* transcripts from stressed and unstressed libraries, Table S2: Putative-Contaminants, Table S3: 437-Clade2-domains, Table S4: 45-Clade2-shared, Table S5: 91-Clade2-PPN, Table S6: Putative-HGT, Table S7: GO-enrichment, and Table S8: Blast-hits-description.
