# Supplementary figures and images for: The Transcriptomes of Xiphinema index and Longidorus elongatus Suggest Independent Acquisition of Some Plant Parasitism Genes by Horizontal Gene Transfer in Early-Branching Nematodes
Source: Genes (Basel). 2017 Oct 23;8(10):287. doi: 10.3390/genes8100287 (PMC5664137; doi:10.3390/genes8100287)

A

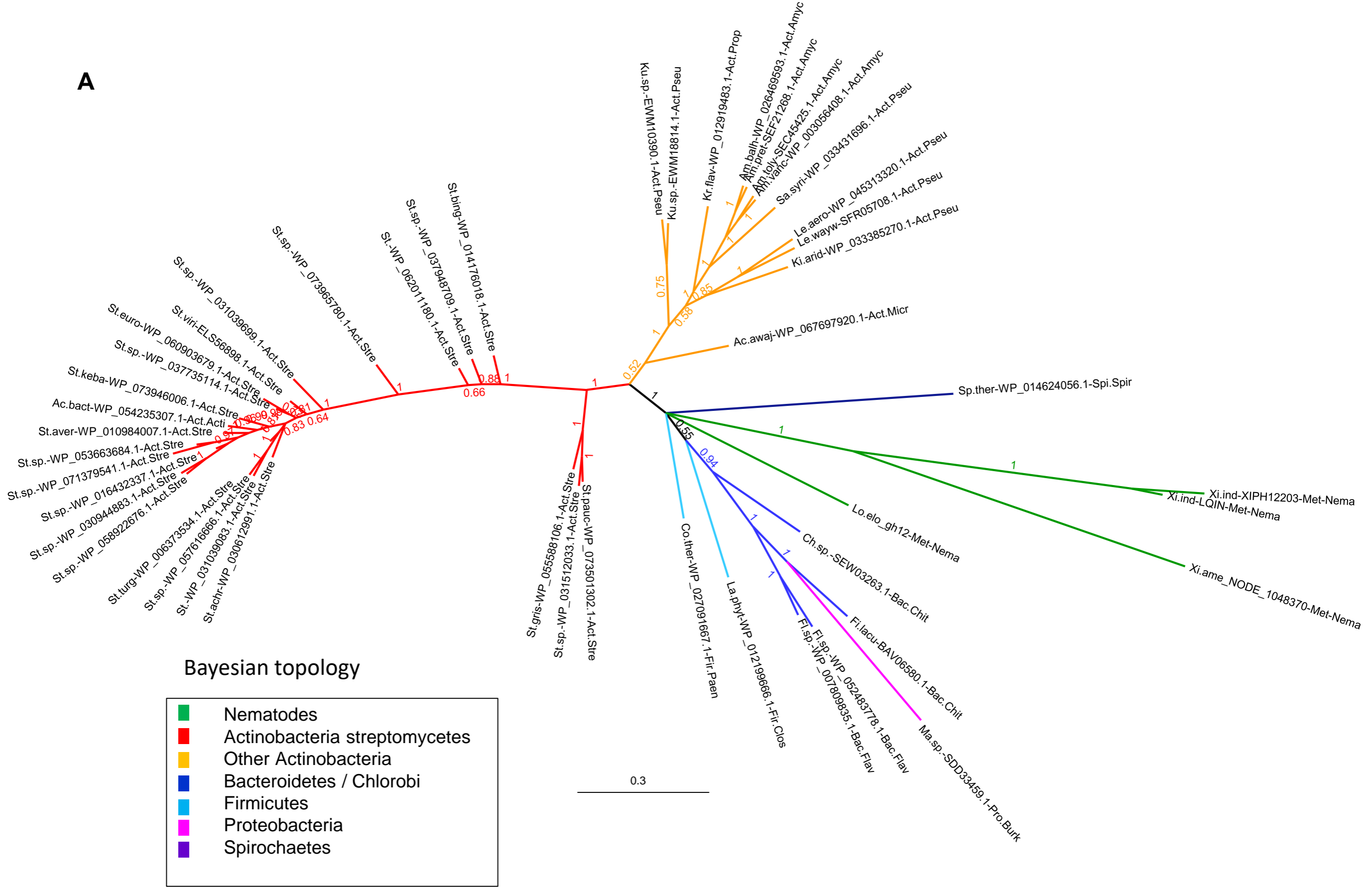

B

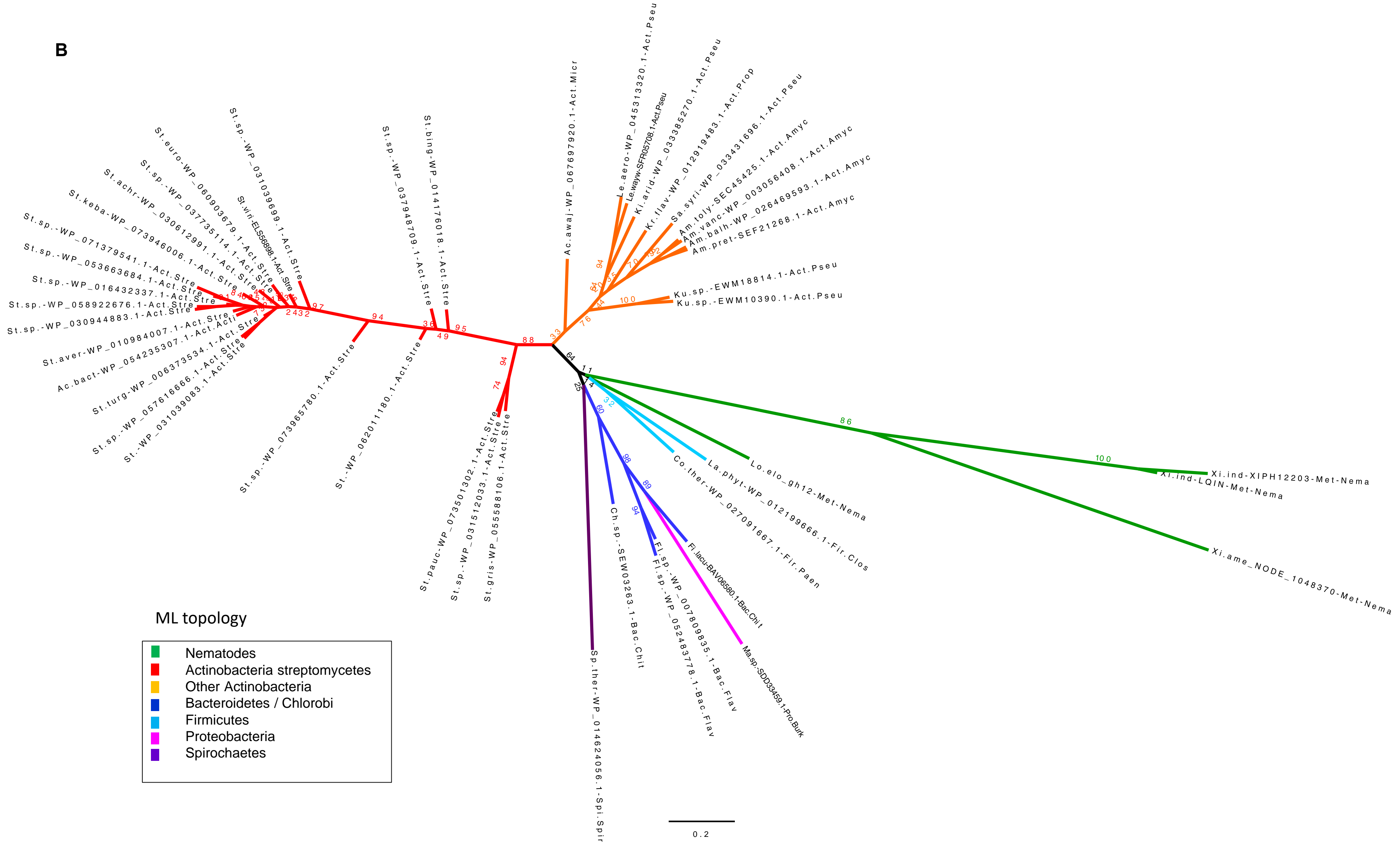

Supplement: Supplementary file 1 [file genes-08-00287-s001.zip › FigureS2-100best-MB-ML.pdf]

B

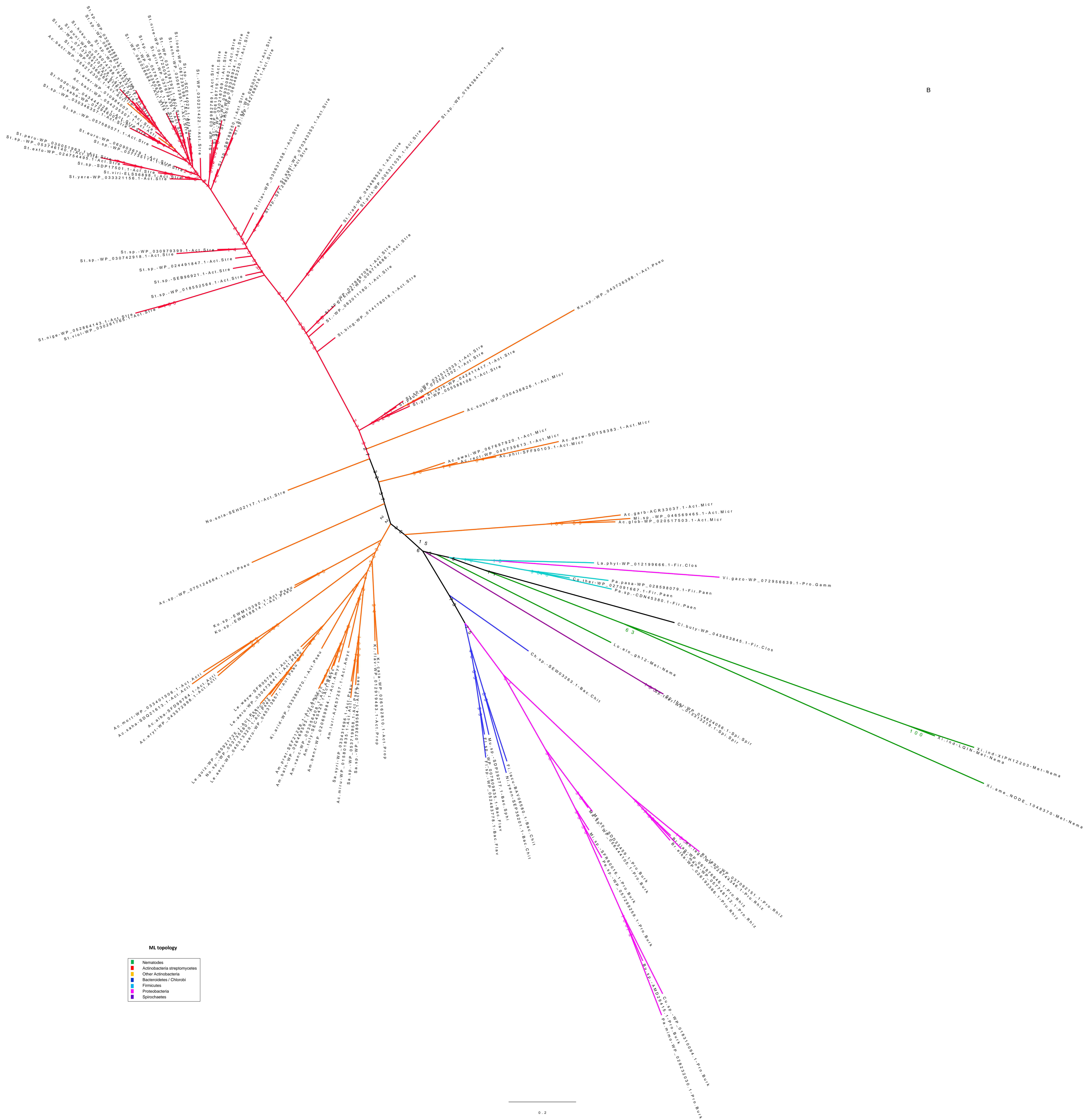

Supplement: Supplementary file 1 [file genes-08-00287-s001.zip › FiguresS1-250best.pdf]
